# Utility of thromboelastography and/or thromboelastometry in adults with sepsis: a systematic review

**DOI:** 10.1186/cc13721

**Published:** 2014-02-10

**Authors:** Marcella C Müller, Joost CM Meijers, Margreeth B Vroom, Nicole P Juffermans

**Affiliations:** Department of Intensive Care Medicine and Laboratory of Experimental Intensive Care and Anesthesiology (L.E.I.C.A.), Academic Medical Center, Room G3-220, Meibergdreef 9, 1105AZ Amsterdam, The Netherlands; Departments of Experimental Vascular Medicine and Vascular Medicine, Academic Medical Center, Amsterdam, The Netherlands

## Abstract

**Introduction:**

Coagulation abnormalities are frequent in sepsis. Conventional coagulation assays, however, have several limitations. A surge of interest exists in the use of point-of-care tests to diagnose hypo- and hypercoagulability in sepsis.

We performed a systematic review of available literature to establish the value of rotational thromboelastography (TEG) and thromboelastometry (ROTEM) compared with standard coagulation tests to detect hyper- or hypocoagulability in sepsis patients. Furthermore, we assessed the value of TEG/ROTEM to identify sepsis patients likely to benefit from therapies that interfere with the coagulation system.

**Methods:**

MEDLINE, EMBASE, and the Cochrane Library were searched from 1 January 1980 to 31 December 2012. The search was limited to adults, and language was limited to English. Reference lists of retrieved articles were hand-searched for additional studies. Ongoing trials were searched on http://www.controlled-trials.com and http://www.clinicaltrials.gov. Studies addressing TEG/ROTEM measurements in adult patients with sepsis admitted to the ICU were considered eligible.

**Results:**

Of 680 screened articles, 18 studies were included, of which two were randomized controlled trials, and 16 were observational cohort studies. In patients with sepsis, results show both hyper- and hypocoagulability, as well as TEG/ROTEM values that fell within reference values. Both hyper- and hypocoagulability were to some extent associated with diffuse intravascular coagulation. Compared with conventional coagulation tests, TEG/ROTEM can detect impaired fibrinolysis, which can possibly help to discriminate between sepsis and systemic inflammatory response syndrome (SIRS). A hypocoagulable profile is associated with increased mortality. The value of TEG/ROTEM to identify patients with sepsis who could possibly benefit from therapies interfering with the coagulation system could not be assessed, because studies addressing this topic were limited.

**Conclusion:**

TEG/ROTEM could be a promising tool in diagnosing alterations in coagulation in sepsis. Further research on the value of TEG/ROTEM in these patients is warranted. Given that coagulopathy is a dynamic process, sequential measurements are needed to understand the coagulation patterns in sepsis, as can be detected by TEG/ROTEM.

## Introduction

Coagulopathy is highly prevalent in sepsis patients and is associated with increased mortality [1]. Coagulopathy results from an imbalance between activation of coagulation and impaired inhibition of coagulation and fibrinolysis. The disturbance between components of the coagulation system leads to a variable clinical picture, ranging from an increased bleeding tendency due to consumption of coagulation factors and platelets, to hypercoagulopathy with disseminated intravascular coagulation (DIC) and (micro-) vascular thrombosis.

Assessment of coagulation status in these patients is complex. Global coagulation tests activating partial thromboplastin time (APTT) and prothrombin time (PT) are used clinically. However, their ability to reflect *in vivo* hypocoagulability accurately is questioned [2]. Also, APTT and PT reflect only a part of the coagulation system and do not provide information on the full balance between coagulation and anticoagulation. Activation of coagulation can be assessed by thrombin generation, but this test is not widely available. Impaired function of the anticoagulant system can be diagnosed by measuring plasma levels of naturally occurring anticoagulant factors antithrombin (AT), protein C, protein S, and tissue factor pathway inhibitor (TFPI). However, these are not readily available for clinical use. The same applies to markers of the activity of the fibrinolytic system [2]. Although activation of the fibrinolytic system can be detected by increased levels of D-dimers and other fibrin-degradation products, specificity is limited [2].

**Figure 1:**
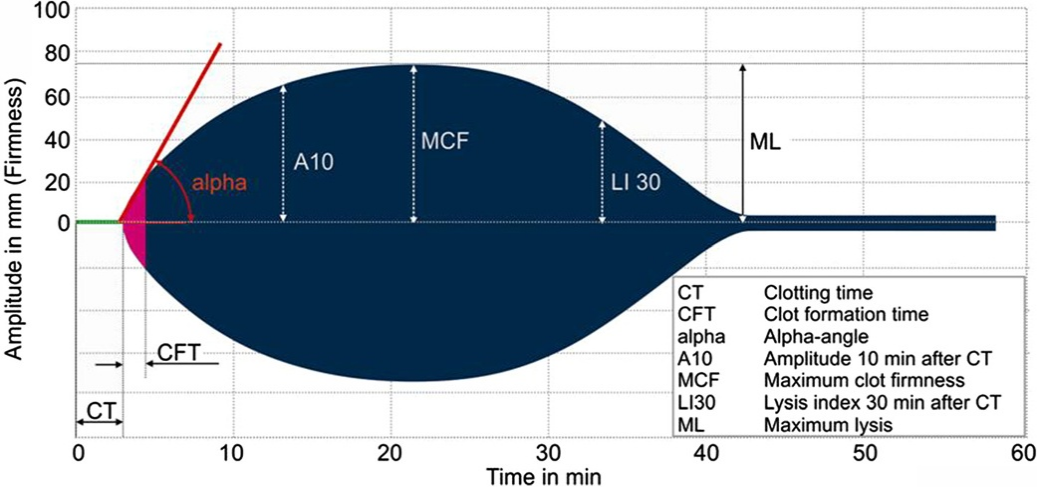
**ROTEM trace with major parameters.** Reference: http://www.rotem.de.

Rotational thromboelastography (TEG) and thromboelastometry (ROTEM) are point-of-care tests, which evaluate whole-clot formation and dissolution. The thromboelastogram arises through movement of the cup (TEG) or the pin (ROTEM). As fibrin forms between the cup and the pin, this movement is influenced and converted to a trace reflecting different phases of the clotting process. Major parameters are reaction time (R) or clotting time (CT), which is the period from the initiation of the test until the beginning of clot formation (Figure 1). *K-*time or clot formation time (CFT) is the period from the start of the clot formation until the curve reaches an amplitude of 20 mm. Kinetics of fibrin formation and cross-linking are expressed by the α-angle, which is the angle between the baseline and the tangent to the TEG/ROTEM curve amplitude. Clot strength is represented by the maximal amplitude of the trace. The degree of fibrinolysis is reflected by the difference between the maximal amplitude and the amplitude measured after 30 and/or 60 minutes (Figure 1). To describe these viscoelastic changes, both systems have their own terminology (Table 1).

**Table 1:** **Parameters displayed on TEG and ROTEM**

	TEG	ROTEM
Time to initial fibrin formation (to 2-mm amplitude)	*R*	CT
Clot strengthening, rapidity of fibrin buildup	*K*	CFT
	α	α
Clot strength, represents maximum dynamics of fibrin and platelet bonding	MA	MCF
Clot breakdown, fibrinolysis at fixed time	CL30, CL60	LI30, LI45, LI60

Reference: http://www.rotem.de and http://www.haemoscope.com.

The technique was developed in the 1940s, but clinical application has been limited. However, technical developments have led to standardization and improved reproducibility of the method [3, 4]. TEG/ROTEM may facilitate diagnosis of clotting abnormalities in sepsis, including hypercoagulable states such as DIC. Other potential advantages could be a more tailor-made administration of therapies that interfere with the coagulation system [5–8]. These tests also may improve prognostication of sepsis [9, 10].

The main research questions for this systemic review were as follows. Can TEG/ROTEM detect sepsis-induced coagulopathy? Is TEG/ROTEM of additional value compared with standard coagulation tests to detect hyper- or hypocoagulability in sepsis patients? Can TEG/ROTEM help to identify sepsis patients likely to benefit from therapies that interfere with the coagulation system (for example, activated protein C, antithrombin, heparin)? We defined our population as critically ill adults with sepsis and TEG/ROTEM as the intervention. Standard coagulation tests, including APTT, PT, INR, and ISTH DIC score, functioned as comparisons. Outcomes of interest were the detection of a hyper- or hypocoagulable state in these patients and the identification of patients likely to benefit from therapies affecting the coagulation system.

## Materials and methods

### Data sources

An electronic search was conducted in MEDLINE, EMBASE, and the Cochrane Library. In addition, we searched for ongoing trials on http://www.controlled-trials.com and http://www.clinicaltrials.gov. We hand-searched the reference lists of retrieved articles, reviews, and editorials for additional studies. Language was limited to articles written in English and published from 1 January 1980 to 31 December 2012. We did not register our protocol.

### Study selection

Two authors (MCM and NPJ) performed the literature search and selected the relevant articles for inclusion. Differences were resolved in consensus meetings. Predefined eligibility criteria were used. Studies were included if TEG/ROTEM measurements were performed in adult patients with sepsis admitted to the ICU. Randomized controlled trials, prospective and retrospective cohorts, and case series were all eligible for inclusion. Reviews, correspondences, case reports, expert opinions, and editorials were excluded. We also excluded all studies conducted outside the ICU or that involved subjects younger than 18 years.

### Data-collection process

Two of the authors (MCM and NPJ) independently extracted the data by using a predefined extraction sheet. Discrepancies were resolved in a consensus meeting. If agreement could not be reached, a third author was consulted (MBV) to resolve disagreement. The extracted data were general methodologic characteristics, setting, characteristics of the study population, used test (ROTEM or TEG), timing of thromboelastography, possible comparison of thromboelastography results with a reference test, administration of therapies interfering with the coagulation system, and main outcomes. Furthermore, possible limitations of each study were listed. No assumptions or simplifications were made.

### Assessment of methodologic quality

We used the QUADAS-2 checklist to assess the quality of diagnostic studies [11, 12]. Studies were assessed for the risk of bias in patient selection, conduct and interpretation of TEG/ROTEM, use and interpretation of a reference standard, and patient flow. For all research questions, methodologic aspects, including the use of a comparison of TEG/ROTEM measurement, the individual studies were assessed. Furthermore, studies were judged with respect to patient population and selection (use of definition of sepsis). Details on how TEG/ROTEM and reference tests were conducted and interpreted (for example, timing of the tests, blinded interpretation) were assessed. Subsequently, quality of evidence was judged and described in accordance with the GRADE approach (high, moderate, low, and very low). Rating of quality of evidence was based on trial design (for example, randomized clinical trial or not), risk of bias and imprecision (for example, patient selection and patient flow, method of conduct and interpretation of TEG/ROTEM, and reference test results). We verified whether results of the retrieved trials and abstracts had been published.

### Definitions

Hypocoagulability can be defined as prolonged CT/*R* and CFT/*K* times and/or decreased MCF/MA and alpha angle [4]. Conversely, a hypercoagulable state can be detected by shortened reaction times (CT/*R* and/or CFT/*K*) and enhanced clot formation, expressed as increased alpha and/or high maximal amplitude (MCF/MA). However, no universal definitions of hypo- and hypercoagulability assessed by TEG/ROTEM exist.

## Results

### Study selection

**Figure 2:**
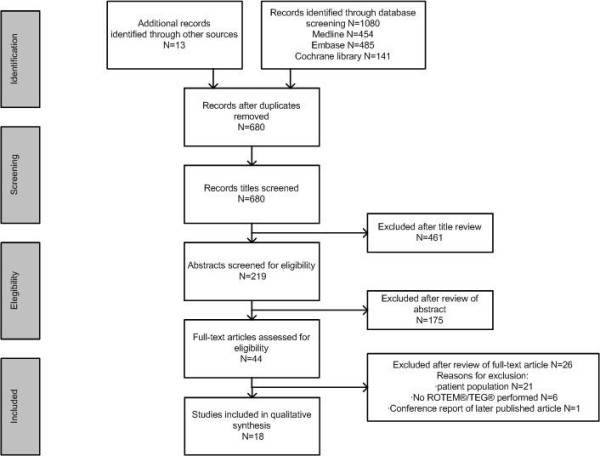
**PRISMA flow diagram.**

Of 680 screened articles, we included 18 studies (Table 2). An overview of the search is presented in Figure 2. Twenty-six studies were excluded because of inappropriate patient population (*N* = 21 [13–33]), no report of TEG/ROTEM data (*N* = 6 [31, 32, 34–37]), and one conference poster [38].

**Table 2:** **Studies assessing TEG/ROTEM in sepsis**

Author, year	Type of study	Population ( ***N*** )	ROTEM or TEG	Timing of measurement	Comparison	Main ROTEM/TEG findings
Gonano [40]	Subanalysis of randomized controlled trial	Severe sepsis (n = 33)	TEG	At diagnosis and daily thereafter.	PT, APTT, AT	All patients were hypercoagulable (shortened R and CT, increased α and MA). Antithrombin treatment did not affect TEG values.
Raineri (abstract) [39]	Randomized controlled trial	Severe sepsis and septic shock (*n* = 16)	TEG	Daily for 2 weeks and day 17, 20, 23, 28	PAI-1	In patients without tight glycemic control (TGC), fibrinolysis was decreased (increased lysis index and increased PAI-1), compared with sepsis patients not treated with TGC.
Collins [43]	Prospective observational	Sepsis (*n* = 38), healthy controls (*n* = 32)	ROTEM	Not stated	PT, APTT fibrinogen, factor levels	In sepsis, there was delayed activation of hemostasis, once activated clot formation was exaggerated (increased MCF, α angle, area under clot firmness curve)
Chiari (abstract) [47]	Prospective observational	Severe sepsis (n = 15)	ROTEM	Before and first day of treatment with activated protein C	APTT, PT	Only CT significantly increased with activated protein C treatment
Daudel [44]	Prospective cohort	Sepsis (*n* = 30)	ROTEM	0-48 hours after diagnosis and at discharge	INR, APTT, fibrinogen, individual factors	All parameters within reference values. Patients with SOFA >10 had increased coagulation (reduced MCF and alpha and increased CFT).
Schmittinger (abstract) [46]	Prospective observational	Severe sepsis (n = 49), postoperative SIRS (n = 27)	ROTEM	Day 1, 4, 7 after admission	None	All parameters within reference values. Mortality 58.3% in patients with signs of hypocoagulation vs. 9.1% in those with signs of hypercoagulability.
Sivula [41]	Prospective observational	Severe sepsis (n = 28), healthy controls (n = 8)	ROTEM	Day 1	APTT, AT, D-dimer, fibrinogen	Only sepsis patients with DIC were hypocoagulable compared to healthy controls. CFT, alpha and MCF discriminated well between DIC and non- DIC. Decreased fibrinolysis in all sepsis patients versus controls.
Adamzik [50]	Prospective observational	Sepsis (n = 56), postoperative controls (n = 52)	ROTEM	Within 24 hours of sepsis diagnosis	Procalcitonin, IL-6, CRP	Increased lysis index in sepsis compared to postoperative controls (97 ± 0.3% vs. 92 ± 0.5%, p < 0.001). CFT, alpha and MCF did not differ between groups. Lysis index had best accuracy for diagnosis sepsis.
Altmann [48]	Prospective observational	Septic shock (n = 16), severe sepsis (n = 7), SIRS (n = 10)	ROTEM	0, 12, 24, 48 h after inclusion	None	All parameters within reference values.
Durila [49]	Prospective observational	Severe sepsis (n = 44)	TEG	Not stated	INR, APTT, fibrinogen, AT	All parameters within reference values.
Adamzik [9]	Prospective observational	Sepsis (n = 98)	ROTEM	Within 24 hours of diagnosis	INR	39% of sepsis patients had normal CFT, MCF, and α angle, values in 61% with pathologic variable showed broad distribution Hypocoagulable profile associated with increased mortality (OR 4.1; 95% CI 1.4-11.9).
Cortegiani (abstract) [51]	Prospective observational	Severe sepsis (n = 31), postoperative (n = 31)	TEG	Within 12 hours of diagnosis	None	Sepsis patients had lower α angle, other TEG parameters did not differ.
Brenner [54]	Prospective observational	Septic shock (n = 30), major surgery (n = 30), healthy volunteers (n = 30)	ROTEM	Sepsis: at diagnosis, 24 h, 4, 7, 14, 28 days	Prothrombin index, factor levels, IL-6, TNF-α	In sepsis patients, majority of ROTEM analysis within reference values; however, sepsis patients with DIC showed more hypocoagulable traces compared with those without DIC were more hypercoagulable Compared with surgical and healthy controls fibrinolysis was impaired in sepsis patients.
Durila [53]	Prospective observational	Postsurgical esophagectomy (n = 38), of these, nine developed sepsis.	TEG	Morning of surgery and daily day 1-6 post operative	APTT, INR, CRP, lactate, IL-6, procalcitonin, AT, D-dimer	On postoperative day 6, sepsis patients had higher lysis index compared with SIRS patients. Overall TEG not helpful in discriminating sepsis from SIRS
Massion [52]	Prospective cohort	Septic shock (n = 39)	ROTEM	Admission to day 7	APTT, PT, Thrombin generation, factor levels, AT, protein C	Fibrinolysis was decreased (increased lysis indexes), associated with hypocoagulation in conventional coagulation tests (decreased protein C and AT). Other parameters within reference values (CT, MCF and alpha). Nonsurvivors were more hypocoagulable, but ROTEM values were not independently associated with mortality
Ostrowski [10]	Prospective observational	Severe sepsis (*N* = 13) and septic shock (*N* = 37)	TEG	Day 1-4	ISTH DIC score, INR, APTT, D-dimer, fibrinogen, CRP	According to cloth strength (MA), 48% of sepsis patients was normocoagulable, 22% hypocoagulable and 30% hypercoagulable. 50% of patients with hypocoagulable profile had overt DIC, versus none of those with a hypercoagulable profile. Hypocoagulable profile predicts 28-day mortality if corrected for SOFA, but not if corrected for SAPS II score.
Viljoen [42]	Not stated	Sepsis (*n* = 15), trauma (*n* = 14), surgery (*n* = 21), healthy control (*n* = 23)	TEG	Daily	Plasma elastase-α sub 1 PI	Sepsis patients were hypocoagulable compared with surgery patients and controls. Sepsis patients had higher elastase-α sub1 proteinase inhibitor levels compared with controls, without a correlation with TEG parameters.
Umgelter (abstract) [45]	Not stated	Sepsis (*n* = 21), no sepsis (*n* = 23)	ROTEM	Not stated	Thrombin time, D-dimer, AT	ROTEM did not discriminate between septic and nonseptic cirrhosis patients

### Study characteristics

We included two randomized controlled trials [39, 40] and 16 observational studies [9, 10, 41–54]. Of the observational studies, 14 were prospective and one abstract [45] and one article [42] did not state whether the study was prospective or retrospective. Of the included studies, 11 [9, 10, 41, 43, 44, 48–51, 53, 54] used the sepsis and SIRS criteria defined by the Society of Critical Care Medicine Consensus Conference [55]. Seven studies used TEG [10, 39, 40, 42, 49, 51, 53], and 11 used ROTEM [9, 41, 43–48, 50, 52, 54].

### Risk of bias

The risk of bias within studies is summarized in Table 3. Quality assessment revealed that risk of bias of patient selection was low. However, included studies were heterogeneous regarding conduction and interpretation of TEG/ROTEM, and no studies reported whether results of TEG/ROTEM were interpreted with or without knowledge of the used reference test, which leaves the possibility for interpretation bias. However, applicability concerns of the chosen reference tests are low.

**Table 3:** **Summary of risk of bias and applicability concerns for included studies**

Study	Risk of bias			Applicability concerns		
	Patient selection	Conduction and interpretation of TEG/ROTEM	Use and interpretation of reference standard	Patient flow	Patient selection	Reference standard
Gonano *et al.*[40]	-	?	-	?	-	-
Raineri *et al*. [39]	?	?	-	-	-	-
Collins *et al.*[43]	?	?	-	?	-	-
Chiari *et al.*[47]	-	?	-	?	-	-
Daudel *et al.*[44]	-	-	-	?	-	-
Schmittinger *et al.*[46]	-	-	NA	-	-	NA
Sivula *et al.*[41]	-	?	?	?	-	-
Adamzik *et al.*[50]	-	?	?	-	-	-
Altmann *et al.*[48]	-	?	NA	NA	-	NA
Durila *et al.*[49]	-	+	-	-	-	-
Adamzik *et al.*[9]	-	-	-	+	-	-
Cortegiani *et al.*[51]	?	?	NA	-	-	NA
Brenner *et al.*[54]	-	-	-	-	-	-
Durila *et al.*[53]	-	?	?	+	-	-
Massion *et al.*[52]	-	?	-	-	-	-
Ostrowski *et al.*[10]	-	-	-	+	-	-
Viljoen *et al.*[42]	+	?	?	?	+	+
Umgelter *et al.*[45]	+	+	NA	?	+	NA

-, low risk; +, high risk; ?, unclear risk; NA, not applicable.

In addition to individual sources of bias related to design and methods, we identified the lack of information about the conduct and interpretation of the TEG/ROTEM test and results as the most important source of bias across all studies (Table 3). We verified whether abstracts and trial results had been published. None had been published at December 31, 2012. Construction of a funnel plot was not feasible because of characteristics of retrieved studies.

### Synthesis of results

Studies varied widely in the way they were conducted. Main differences were timing of TEG/ROTEM measurements, number of measurements, use of preset reference values or control group to assess derangements in TEG/ROTEM measurements in sepsis, and variable use of different comparison tests. Because of this clinically relevant heterogeneity, we carried out a narrative synthesis of the results of the included studies.

### Ability of TEG/ROTEM to detect sepsis-induced coagulopathy

In five studies, all TEG/ROTEM measurements in sepsis were within reference values [44, 46, 48, 49, 54]. These studies included a total of 176 sepsis and severe sepsis patients. In seven studies, TEG/ROTEM revealed pathologic changes ranging from distinct hypercoagulability [40] to predominantly hypocoagulable profiles [42, 51]. Four prospective observational studies, together including 214 patients, reported heterogeneous results with patients showing hyper- and hypocoagulability [9, 10, 41, 43]. Impaired fibrinolysis in sepsis patients was demonstrated in five different observational studies, with a total of 162 patients [41, 50, 52–54]. Two of these studies reported increased lysis indices as the only abnormal ROTEM parameter in 30 and 39 patients with septic shock [52, 54]. In a small cohort of 16 patients with severe sepsis; patients were randomized to tight glycemic control or conventional glucose levels; strict regulation of glucose levels resulted in enhancement of fibrinolysis, as measured by lysis index with ROTEM [39].

Overall, if sepsis-induced coagulopathy was detected, the proportion of sepsis patients with sepsis-induced coagulopathy that was detected by TEG/ROTEM ranged from 43% to 100% [9, 10, 40, 41].

Altogether, in the majority of studies, TEG/ROTEM was able to detect sepsis-induced coagulopathy. However, changes in parameters were heterogeneous, and study designs varied widely, with a lack of clarity in interpretation of test results. Based on this, the quality of evidence supporting the use of TEG/ROTEM to detect sepsis-induced coagulopathy is considered low.

### Additional value of TEG/ROTEM in sepsis compared with standard coagulation tests

Studies designed to compare conventional coagulation tests directly with TEG/ROTEM for the detection of sepsis-induced coagulopathy were not retrieved.

However, two studies assessed the value of thromboelastography in the detection of DIC. A prospective pilot study in 28 sepsis patients showed that CFT, MCF, and α angle discriminated moderately between overt DIC and no DIC. ROC values were 0.815 (CI, 0.624 to 0.935) for CFT, 0.891 (CI, 0.715 to 0.975) for MCF, and 0.828 (CI, 0.639 to 0.943) for α angle. Combination of CFT, MCF, and α resulted in a sensitivity of 100% and a specificity of 75%, with a positive likelihood ratio of 4.0 and negative likelihood ratio of 0.0 for the diagnosis of DIC [41].

A recent study showed that ROTEM values were within reference values. However, patients with overt DIC had prolonged CFT and reduced MCF compared with those without DIC. ROC curves for MCF in EXTEM (0.806; sensitivity, 79%; specificity, 75%) and MCF in INTEM (0.853; sensitivity, 86%; and specificity, 81%) achieved fairly good results [54].

Interestingly, TEG/ROTEM failed to detect coagulopathy in two observational studies, whereas conventional coagulation assays were outside normal ranges [44, 49]. In 44 sepsis patients, mean INR, D-dimer, and fibrinogen levels were increased, whereas mean TEG values were not [49]. A study of 30 sepsis patients revealed similar results, with decreased levels of individual factor levels and increased APTT tests, whereas ROTEM variables remained within reference values [44].

Some studies addressed the question whether TEG/ROTEM was superior in discriminating sepsis from nonsepsis patients compared with conventional biomarkers. Indeed, two observational studies demonstrated that the lysis index derived from thromboelastometry could be helpful to discriminate between sepsis and postoperative inflammatory response [50, 54]. Decreased fibrinolytic activity, as reflected by the lysis index, was found to discriminate sepsis from postoperative SIRS patients (ROC-AUC, 0.811; sensitivity, 93%; specificity, 50%), which was comparable to CRP and procalcitonin [54]. In a larger cohort of 56 sepsis patients and 52 postoperative controls, lysis index had even better diagnostic value than did procalcitonin (ROC-AUC, 0.901; sensitivity, 84%; and specificity, 94%) [50]. In contrast, in cirrhosis patients and postesophagectomy patients, thromboelastometry variables failed to discriminate between sepsis and nonsepsis patients [45, 53].

### Ability of TEG/ROTEM to identify patients likely to benefit from anticoagulant treatment in sepsis

We hypothesized that TEG/ROTEM might help to identify patients likely to respond to therapies that target coagulopathy. However, we did not find any study addressing this question. The only data on TEG/ROTEM and therapy interfering with coagulation consist of a few small patient series evaluating TEG/ROTEM measurements during anticoagulant treatment. In an observational study of 15 patients treated with rhAPC, ROTEM measurements did not change during treatment [47]. Gonano [40] showed distinct hypercoagulability in 33 patients with severe sepsis, of whom 17 were treated with antithrombin. In these patients, hypercoagulability was not reversed by the treatment.

### Use of TEG/ROTEM in prognostication of outcome

Although initially not looked for, we extracted a limited number of studies that addressed the value of TEG/ROTEM in predicting outcome in sepsis and decided *post hoc* to add these data to the review. In a cohort of 98 sepsis patients, by using multivariate analysis, a hypocoagulable profile on admission was shown to be an independent risk factor for 30-day mortality (OR, 4.1; 95% CI, 1.4 to 11.9) [9]. However, not all studies have unequivocally showed the prognostic value of hypocoagulability with mortality after correcting for disease severity [10, 52].

In 50 patients with severe sepsis, hypocoagulable TEG MA at admission was an independent predictor for 28-day mortality in a multivariate model including SOFA score (hazard ratio, 4.29 (1.35 to 13.65); *P* = 0.014), but not in a model using SAPS II score (hazard ratio, 2.32 (0.66 to 8.15); *P* = 0.188) [10]. Of note, in a multivariate model a hypocoagulable profile due to a persistent deficit in thrombin generation was a strong predictor of hospital mortality (*P* = 0.024), as was APTT (*P* = 0.007) [52]. The presence of hypercoagulability did not predict outcome.

Quality of evidence of studies addressing the value of TEG/ROTEM to predict mortality is considered of moderate quality. Of note, in these studies, most patients had thromboelastography values outside reference ranges.

## Discussion

This systematic review on studies performing TEG/ROTEM measurements in sepsis patients shows that studies were heterogeneous in design, use of control groups, timing of TEG/ROTEM measurements, and chosen end points. Internal validity of most studies is limited. Although most studies included sepsis patients according to the ACCM/SCCP definition, external validity is limited because of relatively small patient groups in most studies. Furthermore, standardization of used tests is limited, and most studies had methodologic flaws, which may have resulted in bias. Thereby, the overall quality of evidence on the value of TEG/ROTEM in adults with sepsis is considered low.

Results of TEG/ROTEM measurements in sepsis vary widely across studies and show both hypo- and hypercoagulability [10, 40–43, 46, 51]. This is consistent with the pathophysiology of “consumption coagulopathy” during DIC, in which microvascular thrombi are formed at the expense of a bleeding tendency because of low levels of platelets and coagulation factors [56]. Of note, heterogeneity of results can also be caused by differences in disease severity, as changes were more obvious in severe sepsis patients [40, 41, 46, 51] than in sepsis patients. In addition, variation in the way studies were conducted has probably contributed to differences in outcome.

Interestingly, the degree of hypocoagulation was found to be associated with severity of organ failure [44]. In a study comparing different patient populations, hypocoagulation measured with TEG was most apparent in sepsis patients and associated with a proinflammatory response and organ failure [42]. Timing of measurements may be relevant to these observations, as hypocoagulation was found to be more obvious in the acute phase of sepsis and returned to normal values toward discharge from the ICU [44, 51].

Included studies varied widely with regard to the detection of hypercoagulability, ranging from 30% [10] to 100% [40], which may have resulted from variation in timing as well as in the definition of hypercoagulability. Of note, TEG/ROTEM clearly demonstrated hypercoagulability in models of endotoxemia [57], with a strong correlation with plasma levels of prothrombin fragments F1 + 2 [58, 59].

In addition to hypo- and hypercoagulability, TEG/ROTEM can detect impairment in fibrinolysis, expressed as increased lysis indices. Hypofibrinolysis has been demonstrated in several studies in sepsis patients [41, 50, 52–54], but the clinical relevance of this finding must be determined. Of note, increased lysis indices were shown to be helpful in discriminating sepsis and SIRS patients [50, 53, 54].

TEG/ROTEM has been shown to be promising in diagnosing DIC, and in particular, the combination of various parameters (reaction times, maximum amplitude, and α angle) improves diagnostic value [41, 54]. A score to detect DIC with the use of thromboelastometry has been developed, including prolonged reaction and *K* times and decreased α angle and maximum amplitude. This score was validated in patients with an underlying disease known to be associated with DIC and with an ISTH DIC [60] score of more than 5. However, to date, this score has not been validated in critically ill patients with sepsis, and included studies in this review consisted of relatively small patient groups. Therefore to date, quality of evidence supporting the use of TEG/ROTEM to diagnose DIC is low, and further research is necessary.

Several factors in the way TEG/ROTEM measurements were conducted may have affected the results of included studies. First, coagulopathy in sepsis is a dynamic process, evolving from subtle activation of coagulation to overt DIC. Therefore, timing may greatly influence TEG/ROTEM results. Performing sequential measurements will probably provide better insight into the development of coagulation derangements. Timing and number of measurements in included studies varied widely.

Second, no uniform definitions exist of hypo- and hypercoagulability. Reference values for patients with sepsis are not widely assessed, and only one study determined cut-off values for a cohort of sepsis patients [9]. Some studies classified patients as hypo- or hypercoagulable when measurements were outside preset reference ranges [10, 40, 44, 46]; others compared patients with healthy individuals [41, 43, 50, 52, 54]; and some compared mean or median values among or within different patient groups [9, 39, 42, 45–51, 53, 54]. To compare patient categories and possibly investigate therapeutic interventions in the coagulation system, validated universal reference values and definitions are essential. For ROTEM, a multicenter investigation has been undertaken to assess reference values [4]. A study to verify reference intervals of TEG reagents was recently completed (NCT01357928), and we hope that results will contribute to further standardization of TEG.

Third, the included studies differed in the types of reagents used, which may have considerable effects on the results of the studies. Most studies using ROTEM used tissue factor, after recalcification of the citrated sample, to enhance coagulation [41, 43, 44, 48, 52, 54], although some also performed a nonactivated test (NATEM). Of the studies using TEG, only one study reported the use of kaolin activation [49]; the others only recalcified samples before testing. Of note, correlation between non-kaolin-activated and kaolin-activated thromboelastography has shown to be poor [61]. Furthermore, in some studies, a potential heparin effect was blocked by the addition of heparinase [9, 40, 44, 50, 52, 54], whereas others lacked information on the use of heparinase [10, 42].

In the current review, we included studies using ROTEM and studies using TEG. None of the included studies studied both devices. Some studies that compared both devices in other patient populations showed differences in test results between ROTEM and TEG [62, 63], although not all [64]. Therefore, comparisons of results of studies using different devices should be made cautiously.

A hypocoagulable profile detected by TEG/ROTEM seems to be associated with increased mortality among sepsis patients [9, 10, 46]. One could argue that a hypocoagulable profile merely reflects severity of disease. However, in the study of Adamzik [9], hypocoagulable TEG/ROTEM remained an independent predictor of mortality after correction for severity of disease. These findings are in line with results in a larger cohort of general intensive care patients, in which a hypocoagulable profile at admission was associated with an increased mortality [26]. This relation questions the role of coagulation in inflammatory processes. We speculate that enhanced coagulation during infection is functional, thereby preventing dissemination of bacteria. Thereby, hypocoagulability may facilitate enhanced spread of infection and subsequently mortality [65]. The finding that hypocoagulability is associated with organ failure and is an independent risk factor for mortality underlines the need for further research. Currently, two observational prospective trials in sepsis patients are being conducted (NCT00994877 and NCT00299949) on the value of TEG/ROTEM to diagnose DIC and to predict organ failure in sepsis. Results of these studies may help to determine whether TEG/ROTEM can be used to select specific patient populations who are likely to benefit from therapies aimed at intervention in the coagulation cascade during sepsis.

Our review has limitations, which include the lack of a uniform definition of hypo- and hypercoagulability assessed by ROTEM or TEG, different reference values, differences in control groups, and the heterogeneous study quality. Another limitation is related to our search, in which we may have missed studies published in languages other than English, as well as unpublished data. In addition, we might have missed studies because of the applied date restriction and limitation of our search to three databases.

## Conclusion

A considerable proportion of sepsis patients have an altered coagulation status. An abnormal TEG/ROTEM, in particular hypocoagulability, is prognostic for mortality in the critically ill. Also, hypocoagulability as detected by TEG/ROTEM may aid in diagnosing DIC and hypofibrinolysis. Despite heterogeneity and the limited quality of most included studies, application of TEG/ROTEM seems a promising tool in sepsis. However, given that coagulopathy is a dynamic process, more insight into the kinetics of the coagulation alterations, as diagnosed by TEG/ROTEM, is needed before the general use of TEG/ROTEM to detect hyper- or hypocoagulability and DIC can be advocated.

## Key messages

- Current studies on TEG/ROTEM in patients with sepsis are of heterogeneous quality, but TEG/ROTEM could be a promising tool in diagnosing alterations in coagulation in sepsis.
- Hypocoagulability, as detected by TEG/ROTEM, may aid in diagnosing disseminated intravascular coagulation.
- An abnormal TEG/ROTEM, in particular, a hypocoagulable profile, is prognostic for mortality in the critically ill.
- Further research on the value of TEG/ROTEM in sepsis is warranted, and sequential measurements are needed to understand the coagulation patterns, as can be detected by TEG/ROTEM.

## Abbreviations

APTT: Activated partial thromboplastin time
AT: antithrombin
AUC: area under the curve
CFT: clot-formation time
CT: clotting time
DIC: disseminated intravascular coagulation
ICU: intensive care unit
INR: International Normalized Ratio
ISTH: International Society on Thrombosis and Hemostasis
MA: maximal amplitude
MCF: maximal clot firmness
MOF: multiple organ failure
PAI-1: plasminogen activator inhibitor-1
PT: prothrombin time
rhAPC: recombinant activated protein C
ROC: receiver operating characteristic curve
SIRS: systemic inflammatory response
SOFA: Sequential Organ Failure Assessment Score
TFPI: tissue factor pathway inhibitor.
